# Helminth antigens differentially modulate the activation of CD4^+^ and CD8^+^ T lymphocytes of convalescent COVID-19 patients in vitro

**DOI:** 10.1186/s12916-022-02441-x

**Published:** 2022-06-28

**Authors:** Tomabu Adjobimey, Julia Meyer, Vedrana Terkeš, Marijo Parcina, Achim Hoerauf

**Affiliations:** 1 Institute of Medical Microbiology, Immunology and Parasitology (IMMIP), University Hospital Bonn, Sigmund-Freud-Straße 25, 53105 Bonn, Germany; 2 Unité de Biochimie et de Biologie Moléculaire, Faculté des Sciences et Techniques (FAST), Université d’Abomey-Calavi, Abomey-Calavi, Bénin; 3 Department of Infectiology, General Hospital Zadar, Zadar, Croatia; 4 Bonn-Cologne Site, German Center for Infectious Disease Research (DZIF), Bonn, Germany

**Keywords:** COVID-19, SARS-CoV-2, Helminth antigens, CD4^+^ helper and CD8^+^ T cytotoxic T cells, activation markers, CD137 (4-1BB), CD69

## Abstract

**Background:**

The coronavirus disease 2019 (COVID-19) is a respiratory disease caused by SARS-CoV-2, a recently discovered strain of coronavirus. The virus has spread rapidly, causing millions of death worldwide. Contrary to the predictions, prevalence and mortality due to COVID-19 have remained moderate on the African continent. Several factors, including age, genetics, vaccines, and co-infections, might impact the course of the pandemic in Africa. Helminths are highly endemic in Sub-Saharan Africa and are renowned for their ability to evade, skew, and suppress human immune responses through various immune-modulatory mechanisms. Such effects will likely impact SARS-CoV-2 transmission and disease progression.

**Methods:**

Here, we analyzed in vitro the impact of antigen extracts from three major helminth parasites, including *Onchocerca volvulus*, *Brugia malayi*, *and Ascaris lumbricoides*, on the immune reactivity to SARS-CoV-2 peptides in COVID-19 patients. Activation of CD4^+^ and CD8^+^ T cells was investigated using flow cytometry to monitor the expression of CD137 (4-1BB) and CD69. Cytokine expression, including IL-6, IL-10, IFN-γ, and TNFα, was measured by Luminex in cell culture supernatants.

**Results:**

We observed that helminth antigens significantly reduced the frequency of SARS-CoV-2-reactive CD4^+^ T helper cells. In contrast, the expression of SARS-CoV-2-reactive CD8^+^ T cells was not affected and even significantly increased when PBMCs from COVID-19 patients living in Benin, an endemic helminth country, were used. In addition, stimulation with helminth antigens was associated with increased IL-10 and a reduction of IFNγ and TNFα.

**Conclusions:**

Our data offer a plausible explanation for the moderate incidence of COVID-19 in Africa and support the hypothesis that helper T cell-mediated immune responses to SARS-CoV-2 are mitigated in the presence of helminth antigens, while virus-specific cytotoxic T cell responses are maintained.

**Supplementary Information:**

The online version contains supplementary material available at 10.1186/s12916-022-02441-x.

## Background

The coronavirus disease 2019 (COVID-19) is a respiratory disease caused by SARS-CoV-2, a strain of coronavirus initially identified in December 2019 in Wuhan City in China, which can cause severe illness in humans [1]. The virus has spread rapidly, causing millions of death worldwide. Globally, as of April 27t 2022, 508,827,830 confirmed cases of COVID-19, including 6,227,291 deaths, were reported to the WHO [2]. As the pandemic started, WHO predicted millions of COVID-19 deaths in Africa. Contrary to the predictions, 2 years after the first cases, prevalence and mortality have remained surprisingly moderate on the continent [3]. While this has been widely attributed to the younger average age on the continent or the lack of testing capacities, another potential factor might be the co-endemicity of helminths. Indeed, helminth infections, widely spread in Sub-Saharan Africa, are long-lived parasites and renowned for their ability to suppress inflammatory immune reactions to assure persistence in their hosts [4, 5]. Helminths trigger a modified T helper (Th)2 response, where pro-inflammatory components are balanced by regulatory mechanisms including regulatory T and B cells, anti-inflammatory cytokines (IL-10, TGF-β), alternatively activated macrophages, and non-cytolytic antibodies [6–9]. Clinical manifestations in SARS-CoV-2-infections range from asymptomatic to the devastating acute respiratory distress syndrome (ARDS), where the patients require invasive mechanical ventilation [10]. These severe COVID-19 manifestations have been associated with immunological hyperreactivity characterized by a cytokine storm with high levels of pro-inflammatory cytokines like IL-2, IL-6, IFNγ, and TNFα [11, 12]. Previous data have demonstrated that helminths possess significant immunomodulatory properties that dampen harmful hyperinflammatory responses to viruses, bacteria, and other parasites [13–19]. In this context, helminth-mediated immune modulation can potentially impact the physiopathology of SARS-CoV-2 infections [20]. Recent reports suggest an inverse correlation between the incidence of COVID-19 and parasitic infections [21]. Following infection with a pathogenic agent, foreign antigens are processed and presented on the surface of antigen-presenting cells with major histocompatibility complex (MHC) molecules to activate T cell receptors [22]. Activated T cells can differentiate into a heterogeneous population of effector T cells, including CD4^+^ and CD8^+^ T cells that can mediate pathogen clearance [23]. CD8^+^ T cells are one of the main components of immunity against intracellular pathogens such as viruses. In contrast, CD4^+^ T cells are known to help other cells, including cytotoxic T cells and B cells [24, 25]. Upon antigenic activation, T cells upregulate the expression of different markers, including CD69 and CD137 (4-1BB), known as activation-induced markers (AIM). CD137 is a costimulatory member of the TNFR family, expressed on activated CD4^+^ and CD8^+^ T cells. CD137 is upregulated 24 h after stimulation on responding T cells regardless of differentiation stage or profile of cytokine secretion [26]. After a peak 24 h after stimulation, CD137 expression gradually declined between 48 and 72 h [26]. CD69 is a membrane-bound, type II C-lectin receptor classically used as an early marker of lymphocyte activation due to its rapid appearance on the surface of the plasma membrane 2–3 h after stimulation [27]. Despite this early apparition, the expression of CD69 peaks between 18 and 24 h after activation before starting to decrease [28]. Emerging data suggest that CD4^+^ and CD8^+^ T cell responses play key roles in controlling SARS-CoV-2 infection and COVID-19 [29–31].

In the present study, we investigate in vitro the putative impact of helminth co-infection on the activation of SARS-CoV-2-specific T cells in COVID-19 patients by monitoring ex vivo the expression of SARS-CoV-2-induced CD69 and CD137 expression on CD4^+^ and CD8^+^ T cells in the presence or absence of helminth antigens. The findings are particularly relevant for developing countries known to be also endemic for helminth parasites.

## Methods

### Study design and clinical characteristics of study participants

The study was conducted between November 2020 and January 2021 and is part of a larger survey. Patients were recruited in Wershofen (Germany) and Zagreb (Croatia). A total of 50 COVID-19 patients (29 men and 21 women) were included in the study. Samples from convalescent COVID-19 donors (*N* = 6) from Benin, a country endemic for several helminths’ parasites [32–34], were collected at the University Hospital of Cotonou as part of an ongoing survey. None of the study participants were vaccinated against COVID-19. Ethical approval for the study was granted by local ethics committees. All included COVID-19 patients had been reported positive by SARS-CoV-2 RT-PCR in official COVID-19 testing centers. Three (6%) out of the 50 COVID-19 patients had been hospitalized during the acute phase of infection. The majority, 41 (82%), had mild symptoms, whereas 6 (12%) were fully asymptomatic (Table 1). Samples from healthy blood donors were kindly provided by the Haematology Department of the University Hospital of Bonn and were used as controls. Healthy blood donors were all negative at SARS-CoV-2 RT-PCR and serology and included 13 men and 17 women (mean age = 32.5 ± 12.37) (Table 1).

**Table 1:** Epidemiological and clinical characteristics of participants

	Mean age (min–max, mean ± SD) in years	Sex (M/F)	Asymptomatic infection (%)	Mild COVID-19 symptoms (%)	Severe COVID-19 symptoms + hospitalization (%)	Country (Germany/Croatia/Benin)
**CP (*N*** **= 50)**	18–78 (35.08 ± 15.88)	28/22	12	82	6	39/11/0
**HD (*N*** **= 30)**	*18–60 (32.5 ± 12.37)*	13/17	N/A	N/A	N/A	30/0/0
**HEN**	*18-65*	3/3	3	3	0	0/0/6

*CP* COVID-19 patients, *HD* healthy blood donors, *HEN* endemic normals, *N/A* not applicable

### Antigen preparation

Soluble extracts from adult worms of *Onchocerca volvulus*, *Brugia malayi*, and *Ascaris lumbricoides* were prepared as previously described [35]. Briefly, 20 frozen adult worms were thawed and transferred to a Petri dish pre-filled with sterile PBS. Following several washes in PBS, worms were placed inside a glass mortar (VWR, Langenfeld, Germany). Three to 5 ml of RPMI-medium was added, and worms were crushed until a homogenous solution was obtained. The extracts were then centrifuged for 10 min at 300g and 4 °C to remove insoluble material. Supernatants were transferred to a new tube. Protein concentrations were then measured using the Pierce Coomassie Plus (Bradford) Assay Kit (ThermoFisher Scientific, San Diego, CA, USA) according to the manufacturer’s instructions. Aliquots were stored at − 80 °C until use. The optimal concentration for cell stimulation was defined using a titration assay, and the endotoxin level was determined using the Pierce Limulus amoebocyte lysate (LAL) Chromogenic quantification kit (ThermoFisher Scientific). The endotoxin level was routinely below the detection limit of 0.1 EU/ml. Native Influenza A Antigen (H1N1Ag) and *Plasmodium falciparum* Histidine Rich Protein-2 (PfAg) were obtained from BioRad (Bio-Rad Laboratories GmbH, Feldkirchen, Germany) and used in cell culture at an end concentration of 1 μg/ml.

### PBMC isolation

PBMCs were isolated using a Ficoll gradient as previously described [8]. In brief, 15 ml of heparinized blood was diluted 1:2 in PBS (Gibco, Life Technologies, Carlsbad, USA), transferred on 15 ml of Ficoll (PAN Biotech GmbH, Aidenbach, Germany), and centrifuged for 20 min at 800g, 4 °C and without brake. Cell suspensions were then washed twice, 8 min at 300g, 4 °C, and re-suspended in fetal bovine serum (FBS)-supplemented RPMI 1640 medium (Gibco). The cells were finally counted using the trypan blue (ThermoFisher Scientific) exclusion method and diluted for stimulation and culture.

### Stimulation and cell culture

To test the impact of helminth antigens on the immune reactivity to SARS-CoV-2, PBMCs from COVID-19 patients (*N* = 50) were isolated, and 5 × 10^6^ cells per well were plated and stimulated with SARS-CoV-2-spike-protein-peptide-pools (SPP) (Miltenyi Biotech, Bergisch Gladbach, Germany) at a final concentration of 1 μg/ml, in the presence of culture medium (10% FBS in RPMI) or 10 μg/ml (final concentration) of *Brugia malayi*, *Onchocerca volvulus*, or *Ascaris lumbricoides* extracts. The total volume for each well was adjusted to 250 μl with culture medium (10%FBS in RPMI). Cell culture medium and helminth antigens alone were used as stimulation controls. After 24 h of culture (at 37 °C, 5% CO_2_), 200 μl supernatants were collected and frozen until use, and cells were harvested and prepared for FACS staining.

### FACS staining

All reagents were obtained from ThermoFisher Scientific, and staining was performed according to the manufacturer's instructions. Briefly, cells were harvested and washed with FACS buffer (PBS/2% FBS) at 300 g for 8 min. 1 × 10^6^ cells were then resuspended in 100 μl of FACS buffer and blocked with 1 μl of FC- block (ThermoFisher Scientific) for 15 min. 5 μg/1 × 10^5^ cells of anti-human CD3-PerCP (clone: S4.1-7D6), CD4-eF450 (clone: RPA-T4), CD8-FITC (clone: RPA-T8), CD69-APC (clone: FN50), and CD137-PE (clone: 4B4) were added and the cell suspension was incubated for 30 min at 4 °C. Cells were then washed twice with FACS buffer and fixed in 300 μl PFA (4%). To correct spectral overlaps, fluorescence compensation was done using UltraComp ebeads (ThermoFisher Scientific). Data were acquired using a Cytoflex-S flow cytometer (Beckmann Coulter, Krefeld, Germany) and analyzed with FlowJo v10 software (LLC, Ashland, OR, USA).

### Data analysis

**Fig. 1:**
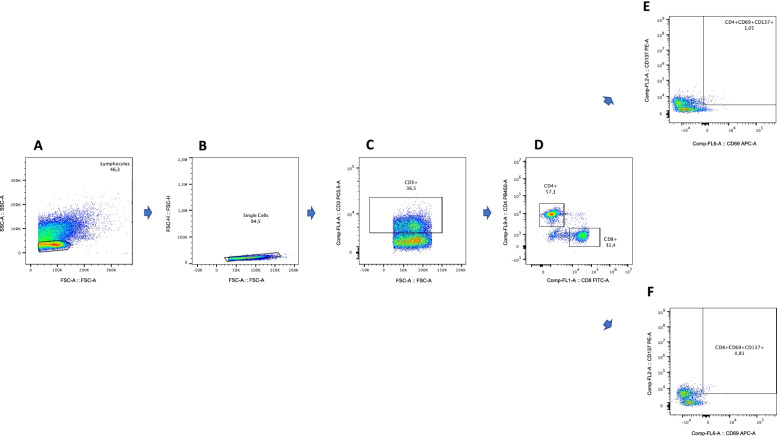
Representative flow cytometry gating strategy for identification of SARS-CoV-2 reactive CD4^+^ and CD8^+^ T cells. Representative dot plots illustrate gating strategy for identification of lymphocytes according to FSC-A and SSC-A (**A**), single-cell gating using FSC-A and FCS-H (**B**), and the gating of CD3^+^ cells according to the expression of CD3 (**C**). The CD3^+^ cell population was used to gate CD4^+^ and CD8^+^ T cell populations (**D**), and finally reactive antigen-specific cells were gated according to the co-expression of CD137 and CD69 in the CD4^+^ (**E**) or CD8^+^ (**F**) populations

The frequency of cells is expressed as the percentage of parent cells (i.e., the frequency of CD4^+^CD69^+^ CD137^+^ T cells is expressed as percentage of the CD4^+^ T cell population). The gating strategy is shown in Fig. 1. Cells were first gated for lymphocytes by forward and side scatter (FSC-A/SSC-A). A single cell gate was set using FSC-A and FCS-H, and then a gate of viable CD3^+^ cells was set and used to gate CD4^+^ and CD8^+^ T cells. Reactive antigen-specific T cells were gated according to the co-expression of CD137^+^ and CD69^+^ in the CD4^+^ and CD8^+^ populations.

### Luminex assay

To quantify cytokine levels in culture supernatants, ProcartaPlex Human Cytokines Panels (ThermoFisher Scientific) were used according to the manufacturer’s instructions. Briefly, anti-IL-6, TNFα, IFNγ, and IL-10-coated magnetic beads were incubated with 25 μl of assay buffer, kit standards, or diluted supernatant (1:2) for 1 h. Twenty-five microliters of biotin-labeled detection antibodies mix was then added. The plates were incubated on an orbital shaker (Stuart, Staffordshire, UK) at 500 rpm for 30 min, and 50 μl of diluted Streptavidin-PE was added. Plates were then incubated for an additional 30 min and washed using a hand-held magnetic plate washer. Afterward, the beads were re-suspended in 120 μl reading buffer. Data were then acquired using a MAGPIX Luminex system and analyzed with ProcartaPlex Analyst software 1.0 (ThermoFisher Scientific).

### Statistics

All graphs were generated using GraphPad Prism 8 (La Jolla, CA, USA). For cell activation data, *P* values were calculated using the Kruskal-Wallis test. Dunn’s multiple comparison test was used to compare all settings. Significance is accepted if *P* < 0.05. For cytokine data, Student’s *t*-test was used for comparisons.

## Results

### Inhibition of the activation of SARS-CoV-2-reactive CD4^+^ T cells by helminth antigens in COVID-19 patients

**Fig. 2:**
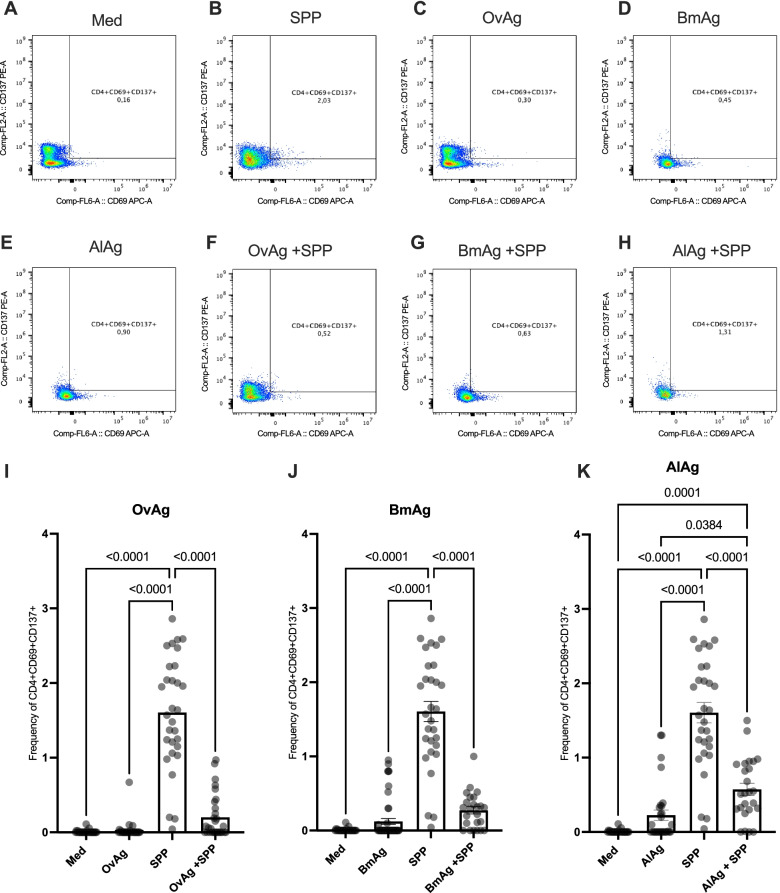
Inhibition of SARS-CoV-2-reactive CD4^+^ T cells by helminth antigens in COVID-19 patients. Dot plots are representative of the frequencies of double positive CD4^+^, expressing CD69 and CD137 after incubation in culture medium (**A**), with SPP (**B**), helminth antigens OvAg (**C**), BmAg (**D**), AlAg (**E**) alone, or SPP in combination with OvAg (**F**), BmAg (**G**), or AlAg (**H**). Graphs summarize the frequencies of CD69^+^ and CD137^+^ in CD4^+^ T cell populations in the different settings (**I**–**K**). Each symbol represents individual donors. Bars indicate means ± SEM of the percentage of SARS-CoV-2-reactive T cells in each setting. Data were obtained from 50 COVID-19 patients

To determine whether and how CD4^+^ helper T cell activation by SARS-CoV-2 peptides is affected by the presence of helminth antigens, we examined the frequency of activated CD4^+^ T cells expressing simultaneously CD69 and CD137 in PBMCs of convalescent COVID-19 patients following stimulation with SARS-CoV-2 peptides alone (SPP), or in combination with helminth antigens (OvAg, BmAg or AlAg). As shown in Fig. 2, the frequency of double-positive CD4^+^ T cells expressing CD69 and CD137 was significantly increased after stimulation with SPP (Fig. 2A, B). In the presence of helminth antigens alone, no significant T cell activation was seen (Fig. 2C–E). The frequency of helper T cells expressing CD69 and CD137 was significantly decreased in PBMCs stimulated with SPP in the presence of helminth antigens (Fig. 2F, G and I–K)*.* Similarly, the helminth antigens also inhibited SARS-CoV-2-specific T cell activation when samples from convalescent COVID-19 patients from helminth endemic region in Benin were used (Additional file 1, Fig. S1).

### No suppression of the activation of SARS-CoV-2-reactive CD8^+^ T cells by helminth antigens in COVID-19 patients

**Fig. 3:**
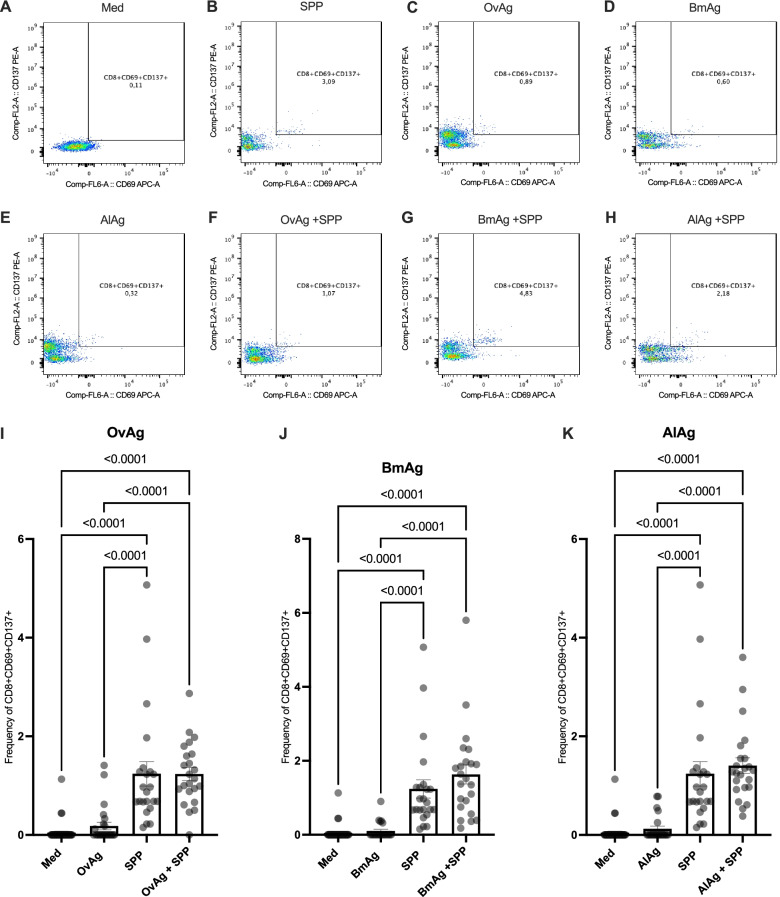
Modulation of SARS-CoV-2-reactive CD8^+^T cells by helminth antigens in COVID-19 patients. Dot plots are representative of the frequencies of the CD8^+^T cells expressing simultaneously the activation markers CD69 and CD137 after incubation in culture medium (**A**), with SPP (**B**), helminth antigens OvAg (**C**), BmAg (**D**), AlAg (**E**) alone or SPP in combination with OvAg (**F**), BmAg (**G**), or AlAg (**H**). Graphs summarize the frequencies of CD69^+^, CD137^+^ in CD8^+^ T cell populations in the different settings (**I**–**K**). Each symbol represents individual donors. Bars indicate Means ± SEM of the percentage of SARS-CoV-2-reactive T cells in each setting. Data were obtained from 50 COVID-19 patients

To determine whether the inhibition of CD4^+^ helper T cells activation also applies to CD8^+^ cytotoxic T cells, we investigated the expression of CD69^+^CD137^+^ in the CD8^+^ T cell population. SPP induced, as described above, a significant increase in the activation of SARS-CoV-2-specific cytotoxic T cells (Fig. 3A, B). Helminth antigens OvAg (Fig. 3C), BmAg (Fig. 3D), and AlAg (Fig. 3E) induced no significant increase in the expression of CD69 and CD137 double-positive CD8^+^ T cells. In contrast to the observations in the CD4^+^ T cell population, the frequency of SARS-CoV-2-reactive CD8^+^ T cells was not affected by the presence of helminth antigens (Fig. 3F–H and I–K). More interestingly, helminth antigens induced a significant increase of SARS-CoV-2-specific CD8^+^ T cells when PBMCs from convalescent COVID-19 patients from endemic helminth region in Benin were used (Additional file 2: Fig. S2).

### Modulation of SARS-CoV-2-reactive CD4^+^ and CD8^+^ T cells is associated with an increase of IL-10

**Fig. 4:**
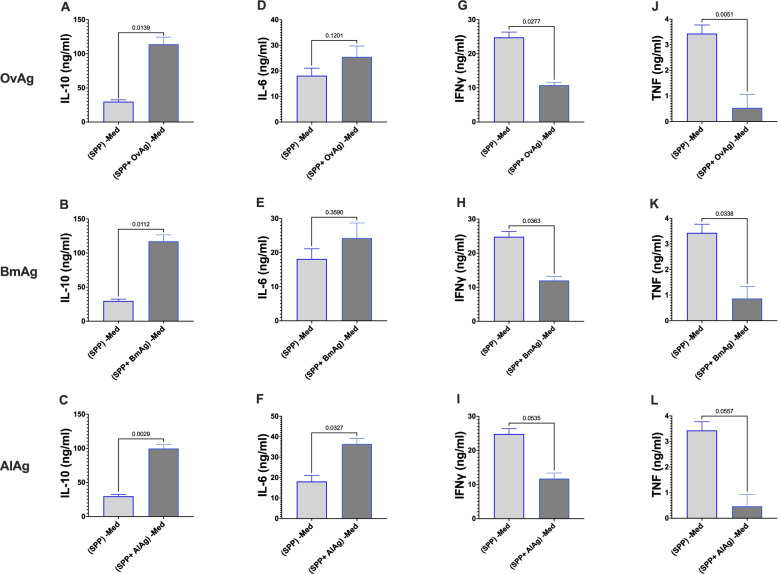
Suppression of SARS-CoV-2 reactive CD4^+^ T cells by helminth antigens correlated with an increase of IL-10 production. Graphs represent cytokine levels of 3 independent experiments, and bars represent means ± SEM of IL-10 (**A**–**C**), IL-6 (**D**–**F**), IFN γ (**G**–**I**), and TNF (**J**–**L**) expression - medium control in the presence of SPP or SPP+ helminth antigens (OvAg, BmAg or AlAg). Data were obtained from 50 COVID-19 patients

To explore the mechanisms associated with this immune suppression by helminth antigens, we analyzed cytokine expression in culture supernatants using a Luminex-based multiplex immunoassay. Indeed, in wells stimulated with SPP in the presence of OvAg, BmAg, or AlAg, significantly higher levels of IL-10 were detected compared to wells stimulated SPP alone (Fig. 3A–C). Interestingly, while a non-significant increase of IL-6 was seen in the presence of OvAg and BmAg, significance was reached in the presence of AlAg (Fig. 4D–F). Moreover, a significant reduction of SPP-induced IFNγ and TNFα expression was seen in the presence of OvAg or BmAg, while a robust trend was observed in the presence of AlAg (Fig. 4G–L).

### No impact of H1N1 and Plasmodium falciparum antigens on the frequencies of SARS-CoV-2-reactive CD4^+^ and CD8^+^ T cells in COVID-19 patients

**Fig. 5:**
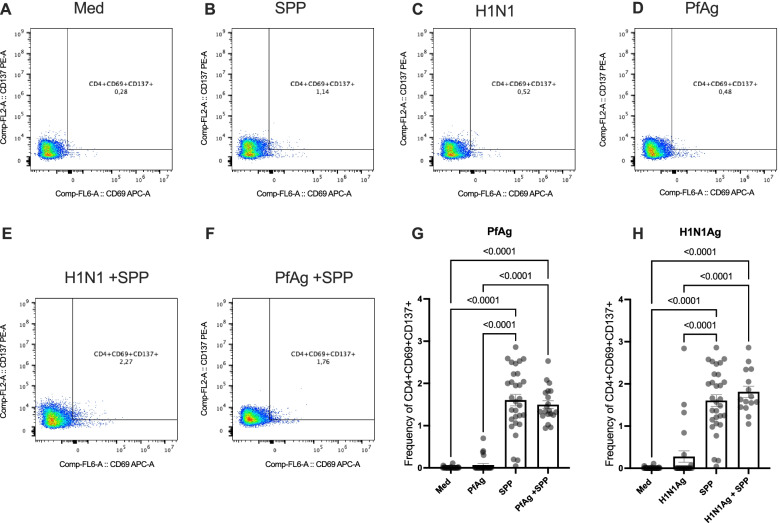
No impact of H1N1Ag or PfAg on SARS-CoV-2-reactive CD4^+^ T cells in COVID-19 patients. Dot plots are representative of the frequencies of double positive CD4^+^ T cells, expressing CD69 and CD137 after incubation in culture medium (**A**), with SPP (**B**), H1N1Ag (**C**), PfAg (**D**), alone or SPP in combination with H1N1Ag (**E**), and PfAg (**F**). Graphs summarize the frequencies of CD69^+^, CD137^+^ in CD4^+^ T cell populations in the different settings in the presence or absence of PfAg (**G**) or H1N1Ag (**H**). Each symbol represents individual donors. Bars indicate Means ± SEM of the percentage of SARS-CoV-2-reactive T cells in each setting. Data were obtained from 15 COVID-19 patients

To determine whether the observed modulation of CD4^+^ and CD8^+^ T cells is specific to helminth antigens, we investigated the effects of Native Influenza A Antigen (H1N1Ag) and *Plasmodium falciparum* Histidine Rich Protein-2 (PfAg) in the same settings. As shown in Fig. 5, H1N1Ag and PfAg alone induced no significant T cell activation (Fig. 5A–D). After co-stimulation with SPP and H1N1Ag on the one hand and SPP and PfAg on the second, neither H1N1Ag nor PfAg significantly affected the expression of SARS-CoV-2-reactive CD4^+^ T helper T cells (Fig. 5E–F and G–H). Similar data were obtained when CD8^+^ T cells were analyzed (Additional file 3: Fig. S3)

## Discussion

Our data support the hypothesis that in the presence of helminth antigens, immune responses to SARS-CoV-2 are mitigated with regard to CD4^+^ T cells and pro-inflammatory cytokine responses. In contrast, SARS-CoV-2-reactive CD8^+^ T cell responses are maintained in COVID-19 patients and even increased when helminth endemic samples were used. This may critically shift the overall immune response to SARS-CoV-2 such that overreaction and severe COVID-19 are avoided, similar to helminths’ ability to mitigate systemic inflammatory response syndrome in animal models and in humans [36, 37].

Even though a role for helminth-induced T cell-intrinsic regulation mechanisms, such as anergy, adaptive tolerance, exhaustion, or plasticity, cannot be excluded, the most probable mechanism for this helminth-induced modulation of T cells might involve innate cells. Indeed, our data indicate that helminth antigens alone do not directly activate T cells. Knowing that CD8^+^ T cells require CD4^+^ T cell help for full activation [38–40], it may seem unexpected that CD8^+^ T cells get activated when CD4^+^ T cells are suppressed. However, even though CD4^+^ T cells are necessary for the development of functional memory CD8^+^ T cells, their requirement for the induction of a primary CD8^+^ T cell response following infection is less clear [23]. Emerging data suggest that Toll-like receptor (TLR) stimulation can bypass the requirement for CD4^+^ T cell help following immunization [41]. These data, together with the fact that helminth antigens are known to activate TLRs [42] offer a possible mechanism for the differential modulation of CD4^+^ and CD8^+^ observed in our study.

The cytokine data suggest that increased IL-10 expression is associated with helminth-induced suppression of antigen-specific CD4^+^ T cell activation. A moderate increase in IL-6 expression was seen only in the presence of AlAg. The differences in IL-6 expression to the controls were, however, not significant, suggesting a key role for IL-10, but no significant part played by IL-6 in the observed modulations.

In our setting, helminth antigens also inhibited SPP-induced expression of Th1 cytokines, including IFNγ and TNFα. The increase of IL-10 might directly induce the suppression of Th1 cytokines, as previously described [43]. Indeed, IL-10 is considered as a potent immune regulatory cytokine [44]. Even though our data did not clarify the origin of IL-10, it can be considered that antigen-presenting cells, chiefly monocytes are the main source of IL-10 in our setting due to the short incubation period. Indeed, monocytes have been defined as the major source of IL-10 in peripheral blood cells [43, 45]. In addition, helminth-induced alternative maturation of monocytes might influence T cell activation [46].

Moreover, IL-10 was shown in other parasitic infections to contribute to the expansion and functional activation of CD8^+^ T cells [47]. In a rodent model, it was demonstrated that antitumor therapy combining IL-10 and oncolytic adenovirus Ad-hTERT increased CD8^+^ T cell-dependent antitumor efficacy [48]. Altogether, these data, in combination with our current finding, suggest a key role for IL-10 in both the suppression of SARS-CoV-2-specific CD4^+^ T cells and the activation or maintenance of CD8^+^ T cells.

Our results with H1N1Ag or PfAg further support the hypothesis that the observed modulation of SARS-CoV-2-reactive CD4^+^ and CD8^+^ T cells in samples from COVID-19 patients is associated with helminth intrinsic immune regulatory properties. Additional data are still required to better define the associated mechanisms.

Our data confirm hypotheses formulated by others, suggesting that helminth parasites and their derivatives might exert an anti-pathological effect in COVID-19 patients [49] and contradict speculations staging helminths as potential potentiators of COVID-19-related morbidity and mortality through suppression of efficient immune response against SARS-CoV-2 [50, 51]. Indeed, despite the implication of Th2 responses in the cytokine storm responsible for the pathology of COVID-19 [51], the typical helminth-induced Th2 immune response is associated with a strong regulatory arm that might significantly mitigate COVID-19 severity [6, 7]. The helminth-induced maintenance or increase of SARS-CoV-2-specific cytotoxic T cells activation observed in samples from COVID-19 patients in our study may be the key element that facilitates the elimination of SARS-CoV-2-infected cells.

The major limitation of the present study is the fact that most samples were collected in Europe. It cannot be excluded that samples from helminth endemic regions react differently. However, samples from 6 convalescent COVID-19 patients obtained from Benin, a country endemic to several helminth parasites [32, 52] were largely in line with the data obtained using European samples. Interestingly, while the helminth antigens did not affect the activation of CD8^+^ T cells in European samples, a significant increase in the activation of CD8^+^ T cells was seen in the Benin samples when PBMCs were stimulated simultaneously with helminth antigens and SARS-CoV-2 peptides.

Even though several other factors might be involved in the low incidence of COVID-19 in helminth endemic countries, and additional data are still required to understand the mechanisms, our current data offer a proof of principle and one plausible explanation for why African countries, endemic for helminth parasites are moderately affected by the COVID-19 pandemic [3]. This differential helminth-induced immunomodulation of CD4^+^ and CD8^+^ T cells might also be relevant to the immunogenicity of current COVID-19 vaccines in endemic helminth regions. Indeed, it is unclear if helminth-induced immunomodulation, as recently hypothesized by Chacin-Bonilla et al. [51], would affect the efficacy of COVID-19 vaccines in helminth endemic regions.

## Conclusions

The present study uniquely demonstrates that antigens from three major helminth parasites (*Brugia malayi*, *Onchocerca volvulus*, and *Ascaris lumbricoides*) could potentially modulate the immune reactivity to SARS-CoV-2 peptides by suppressing reactive CD4^+^ helper T cells while maintaining SARS-CoV-2 reactive CD8^+^ cytotoxic T cells. This finding offers a plausible explanation for the moderate incidence of COVID-19 in helminth endemic countries and opens new research avenues into the impact of helminth-induced immune-regulation on the COVID-19 disease outcome and vaccination effectiveness in tropical countries.

## Supplementary Information

**Additional file 1: Fig. S1**. Inhibition of SARS-CoV-2-reactive CD4^+^ T cells by helminth antigens in COVID-19 patients from a helminth endemic region. Graph summarizes the frequencies of CD69^+^ and CD137^+^ in CD4^+^ T cells in the different settings. Each symbol represents individual donors. Bars indicate Means ± SEM of the percentage of SARS-CoV-2-reactive T cells. Data were obtained from 6 COVID-19 patients in Benin.

**Additional file 2: Fig. S2**. Enhancement of the activation of SARS-CoV-2-specific CD8^+^ T cells by helminth antigens in COVID-19 patients from a helminth endemic region. Graph summarizes the frequencies of CD69^+^ and CD137^+^ in CD8^+^ T cell populations in the different settings. Each symbol represents individual donors. Bars indicate Means ± SEM of the percentage of SARS-CoV-2-reactive T cells in each setting. Data were obtained from 6 COVID-19 patients in Benin.

**Additional file 3: Fig. S3**. No modulation of SARS-CoV-2-specific CD8^+^ T cells by helminth antigens in COVID-19 patients. Graphs summarize the frequencies of CD69^+^ and CD137^+^ in CD8^+^ T cell populations in the different settings in the presence of H1N1Ag (A) or PfAg (B). Each symbol represents individual donors. Bars indicate Means ± SEM of the percentage of SARS-CoV-2-reactive T cells in each setting. Data were obtained from 15 COVID-19 patients.

## Data Availability

All data used for the study are included in the manuscript and the supplementary materials. Additional data related to the work may be available upon request.

## Abbreviations

Ad-hTERT: Adenovirus-human telomerase reverse transcriptase
AIM: Activation-induced markers
AlAg: *Ascaris lumbricoides* antigen
APC: Allophycocyanin
ARDS: Acute respiratory distress syndrome
BmAg: *Brugia malayi* antigen
CD: Cluster of differentiation
COVID-19: Coronavirus disease 2019
eF450: eFluor 450
FACS: Fluorescence-activated cell sorting
FBS: Fetal bovine serum
FC- block: Fragment crystallizable region blocking solution
FITC: Fluorescein isothiocyanate
FSC- (A/H): Forward scatter-(area/height)
H1N1Ag: Flu virus H1N1 (hemagglutinin1, neuraminidase1) antigen
IFNγ: Interferon gamma
IL-: Interleukin-
LAL: Limulus amoebocyte lysate
MHC: Major histocompatibility complex
ml: Milliliter
OvAg: *Onchocerca volvulus* antigen
PBMC: Peripheral Blood Mononuclear Cell
PBS: Phosphate-buffered saline
PE: Phycoerythrin
Perk: Peridinin-chlorophyll-protein
PFA: Paraformaldehyde
PfAg: *Plasmodium falciparum* antigen
rpm: Rotation per minute
RPMI: Roswell Park Memorial Institute
RT-PCR: Real-time PCR
SARS-CoV-2: Severe acute respiratory syndrome coronavirus type 2
SEM: Standard error of mean
SPP: SARS-CoV-2-Spike-protein-peptide-pools
SSC-(A/H): Side scatter-(area/height)
TGF-β: Transforming growth factor-beta
Th1/2: T helper1/2
TLR: Toll-like receptor
TNF: Tumor necrosis factor
TNFR: Tumor necrosis factor receptor
WHO: World Health Organization
