# Supplementary figures and images for: Helminth antigens differentially modulate the activation of CD4+ and CD8+ T lymphocytes of convalescent COVID-19 patients in vitro
Source: BMC Med. 2022 Jun 28;20:241. doi: 10.1186/s12916-022-02441-x (PMC9241220; doi:10.1186/s12916-022-02441-x)

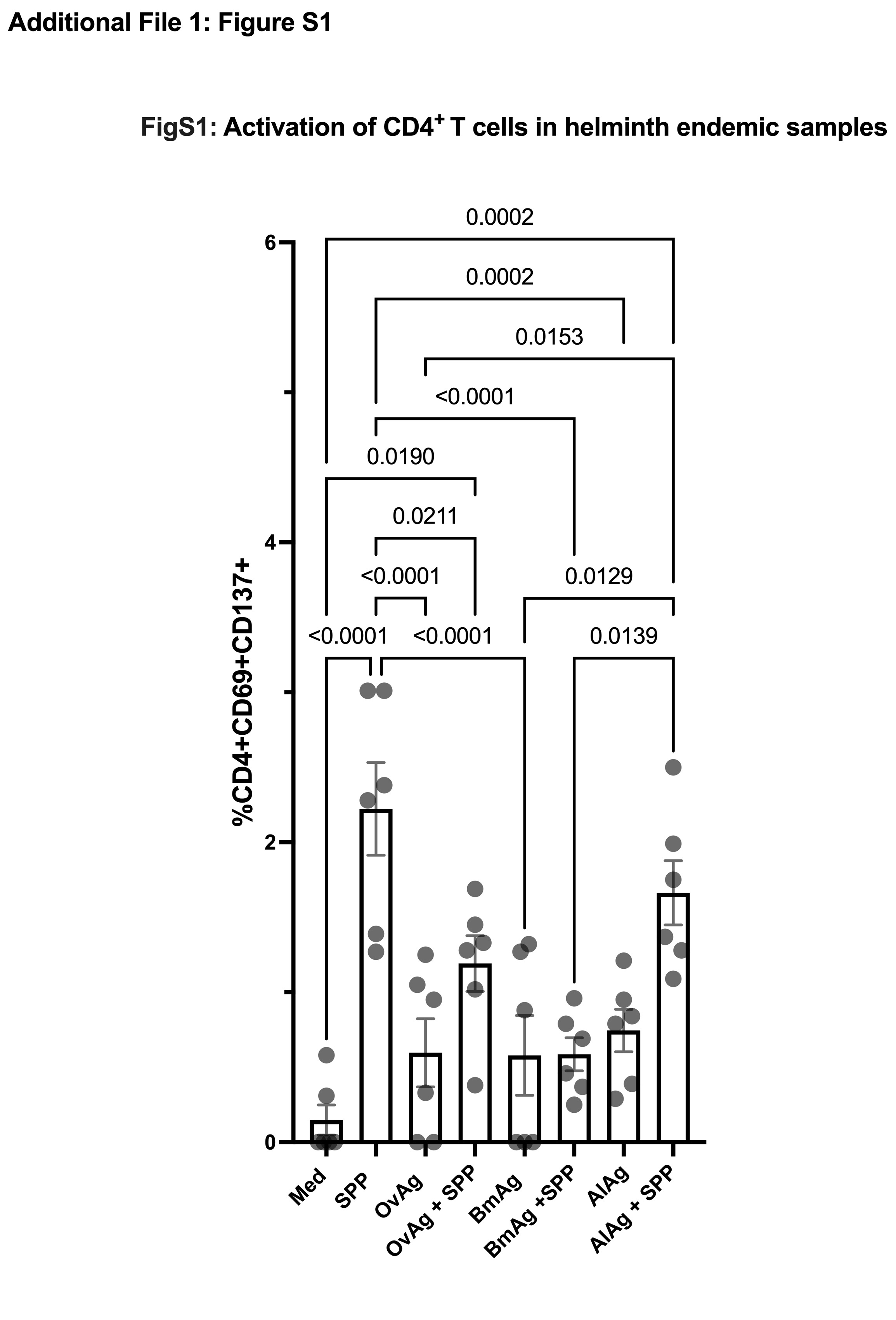

Supplement: Supplementary file 1 — Additional file 1: Fig. S1. Inhibition of SARS-CoV-2-reactive CD4+ T cells by helminth antigens in COVID-19 patients from a helminth endemic region. Graph summarizes the frequencies of CD69+ and CD137+ in CD4+ T cells in the different settings. Each symbol represents individual donors. Bars indicate Means ± SEM of the percentage of SARS-CoV-2-reactive T cells. Data were obtained from 6 COVID-19 patients in Benin. [file 12916_2022_2441_MOESM1_ESM.jpg]

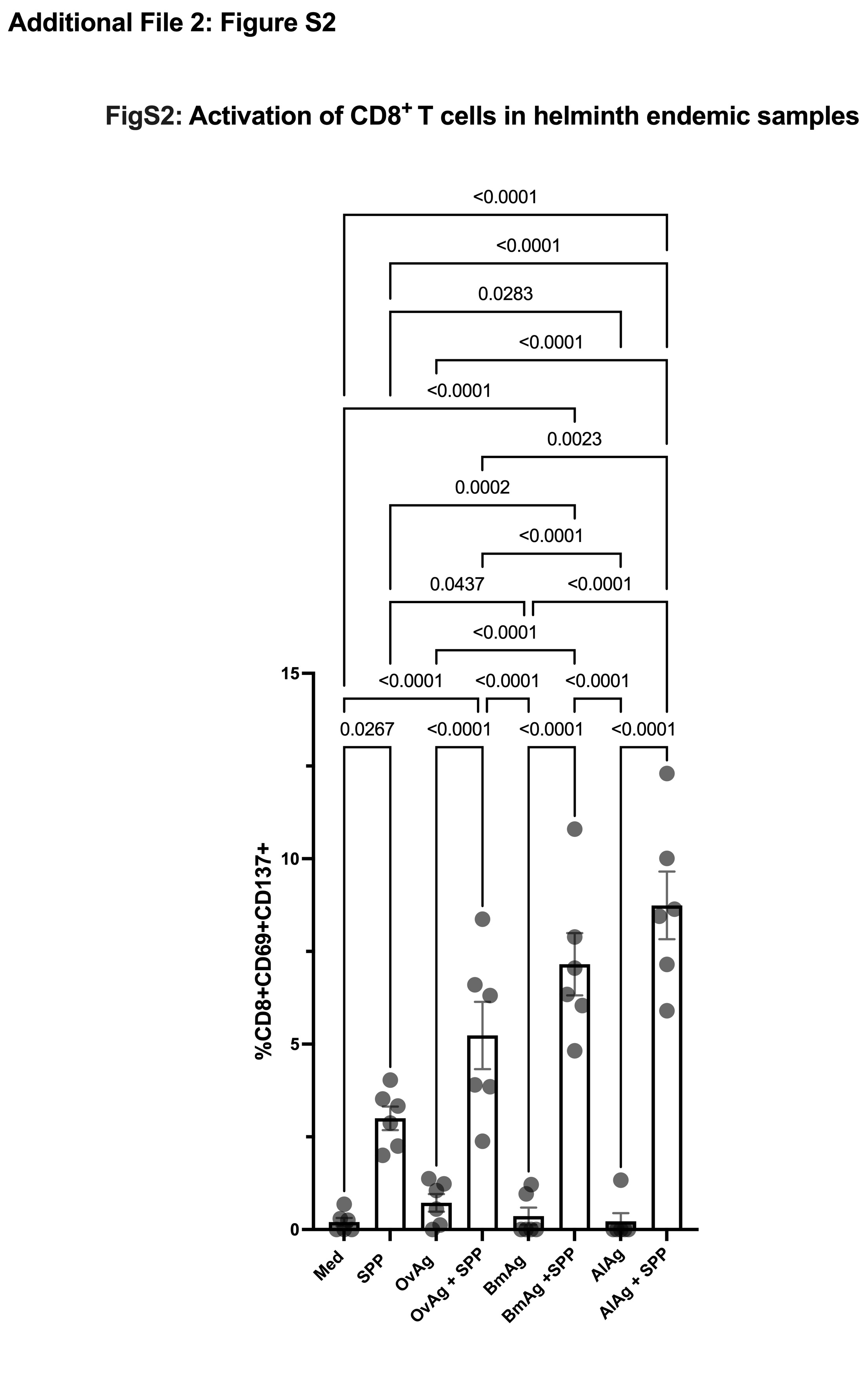

Supplement: Supplementary file 2 — Additional file 2: Fig. S2. Enhancement of the activation of SARS-CoV-2-specific CD8+ T cells by helminth antigens in COVID-19 patients from a helminth endemic region. Graph summarizes the frequencies of CD69+ and CD137+ in CD8+ T cell populations in the different settings. Each symbol represents individual donors. Bars indicate Means ± SEM of the percentage of SARS-CoV-2-reactive T cells in each setting. Data were obtained from 6 COVID-19 patients in Benin. [file 12916_2022_2441_MOESM2_ESM.jpg]

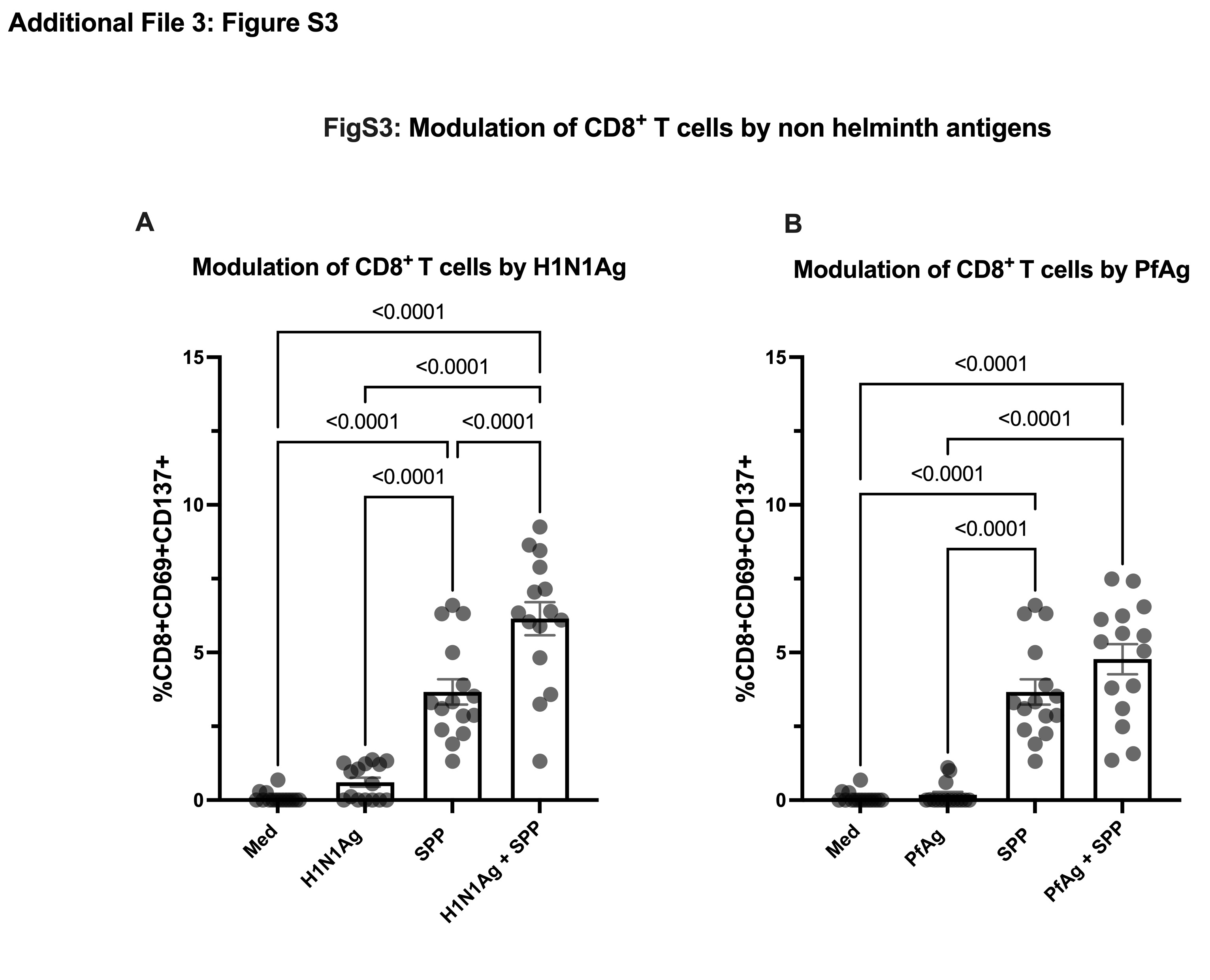

Supplement: Supplementary file 3 — Additional file 3: Fig. S3. No modulation of SARS-CoV-2-specific CD8+ T cells by helminth antigens in COVID-19 patients. Graphs summarize the frequencies of CD69+ and CD137+ in CD8+ T cell populations in the different settings in the presence of H1N1Ag (A) or PfAg (B). Each symbol represents individual donors. Bars indicate Means ± SEM of the percentage of SARS-CoV-2-reactive T cells in each setting. Data were obtained from 15 COVID-19 patients. [file 12916_2022_2441_MOESM3_ESM.jpg]
